# In silico prediction of immune-escaping hot spots for future COVID-19 vaccine design

**DOI:** 10.1038/s41598-023-40741-1

**Published:** 2023-08-18

**Authors:** Sing-Han Huang, Yi-Ting Chen, Xiang-Yu Lin, Yi-Yi Ly, Ssu-Ting Lien, Pei-Hsin Chen, Cheng-Tang Wang, Suh-Chin Wu, Chwen-Cheng Chen, Ching-Yung Lin

**Affiliations:** 1 Graphen Inc., New York, NY 10110 USA; 2 Adimmune Corp., Taichung City, 427003 Taiwan

**Keywords:** Computational biology and bioinformatics, Immunology

## Abstract

The COVID-19 pandemic has had a widespread impact on a global scale, and the evolution of considerable dominants has already taken place. Some variants contained certain key mutations located on the receptor binding domain (RBD) of spike protein, such as E484K and N501Y. It is increasingly worrying that these variants could impair the efficacy of current vaccines or therapies. Therefore, analyzing and predicting the high-risk mutations of SARS-CoV-2 spike glycoprotein is crucial to design future vaccines against the different variants. In this work, we proposed an in silico approach, immune-escaping score (*IES*), to predict high-risk immune-escaping hot spots on the receptor-binding domain (RBD), implemented through integrated delta binding free energy measured by computational mutagenesis of spike-antibody complexes and mutation frequency calculated from viral genome sequencing data. We identified 23 potentially immune-escaping mutations on the RBD by using *IES*, nine of which occurred in omicron variants (R346K, K417N, N440K, L452Q, L452R, S477N, T478K, F490S, and N501Y), despite our dataset being curated before the omicron first appeared. The highest immune-escaping score (*IES* = 1) was found for E484K, which agrees with recent studies stating that the mutation significantly reduced the efficacy of neutralization antibodies. Furthermore, our predicted delta binding free energy and *IES* show a high correlation with high-throughput deep mutational scanning data (Pearson’s *r* = 0.70) and experimentally measured neutralization titers data (mean Pearson’s *r* = −0.80). In summary, our work presents a new method to identify the potentially immune-escaping mutations on the RBD and provides valuable insights into future COVID-19 vaccine design.

## Introduction

The COVID-19 pandemic has had a widespread impact on a global scale. COVID-19 is an infectious disease caused by the severe acute respiratory syndrome coronavirus 2 (SARS-CoV-2)^1^. SARS-CoV-2 belongs to the betacoronavirus 2B lineage of the coronavirus family^2^. The SARS-CoV-2 spike (S) glycoprotein is a type I membrane protein forming a trimer and consists of two functional subunits^3^. The S1 subunit is responsible for binding to the host cell receptor, whereas the S2 subunit is responsible for the fusion of the viral and host cell membranes^4^. The S1 subunit can further be classified into the N-terminal domain (NTD), receptor-binding domain (RBD), and C-terminal domains^3^. The RBD can exist in two distinct conformations—the open state and the closed state, these conformational changes likely play a crucial role in the function of the protein^5,6^. In general, the RBD tends to exist in a single-RBD-open conformation, however, in certain situations, such as receptor binding or specific mutations in the protein, multi-RBD-open conformations can occur^6–9^. The receptor-binding motif (RBM) is a specific region within the RBD that is exposed when the RBD is in the open conformation and is responsible for binding to cell-surface receptors^10^. The RBD of the S1 protein plays a critical role in the viral infection process. It is responsible for interacting with the ACE2 receptor on the surface of host cells, enabling the virus to attach and enter the cell. Developing effective vaccines and therapeutics is essential to combat the virus. Given the significance of the RBD in viral attachment and entry, it becomes a prime target for designing immunogens^11–13^. Neutralizing antibodies bind predominantly to the RBD of the S1 protein, while some neutralizing antibodies can bind to the S2 domain^9,13–15^. To better understand the mechanisms of SARS-CoV-2 and identify the most effective neutralizing variants, researchers have designed numerous mutations in the spike protein. Mutagenesis studies of various coronaviruses based on structural biology, including MERS-CoV, SARS-CoV, and SARS-CoV-2, have demonstrated that the stability of the prefusion structure is crucial for viral fusion and infection. These mutations are aimed at altering specific regions of the spike protein to study their impact on viral behavior and immune response^7,16,17^.

Since SARS-CoV-2 first appeared, considerable evolution has taken place, including dominant variants of concern (VOCs) defined by the World Health Organization (WHO)^18^, such as alpha, delta, and recent omicron variants. These VOCs have shown evidence of higher transmissibility and immune-escaping ability^19–22^. The VOCs contained certain key mutations, such as E484K and N501Y, that were located on the RBD of the spike protein. Moreover, some mutations also occurred at an antigenic supersite of an N-terminal domain NTD^23,24^. Both RBD and NTD are the targets of potent virus-neutralizing antibodies against the spike protein. As of December 2022, the FDA has approved a total of four COVID-19 vaccines, by the providers Pfizer-BioNTech, Moderna, Janssen, and Novavax. Pfizer-BioNTech and Moderna COVID-19 vaccines are so-called mRNA vaccines, whereas Janssen’s COVID-19 vaccines are viral vector vaccines and Novavax COVID-19 vaccines are protein subunit vaccines^25^. As SARS-CoV-2 continues to evolve and mutate, the arising of new VOCs cannot be prevented, and the alterations of the RBD and NTD in spike protein result in reducing the effectiveness of vaccines that are currently in use. To design effective vaccines, it is crucial to thoroughly understand the mechanisms underlying viral infection and the interactions between the virus and neutralizing antibodies^26^. Therefore, it remains crucial to develop future vaccines to protect against future mutations and variants.

As the number of available virus-antibody co-crystal structures and viral genome sequences in the COVID-19 pandemic rapidly increases, there is an opportunity to develop a fast and accurate computational method to predict high-risk mutations and provide recommendations about the COVID-19 vaccine design of future SARS-CoV-2 spike protein variants. Here, we proposed an integrated computational approach, immune-escaping score (*IES*), in which we implemented through integrated delta binding free energy measured by computational mutagenesis of protein structures (i.e., spike-antibody complexes) and mutation frequency calculated from viral genome sequencing data, to predict high-risk immune-escaping mutations in the spike protein. We observed that the antibodies mainly contacted sites 346 to 517 within the RBD of the spike. To further validate our predicted delta binding free energy, we used the immune-escaping ability data from experimental deep mutational scanning^27,28^, and our predicted results highly correlated (Pearson’s *r* = 0.70) with the high-throughput experimental data. We predicted high-risk mutations for S494R and G485R with high delta binding free energy, which were previously unnoticed immune-escaping mutations. Finally, we integrated the delta binding free energy and the mutation frequency to calculate the *IES* to predict the potentially immune-escaping hot spots. Here, we identified 23 hot spots on the RBD that had both a high delta binding free energy and a certain degree of mutation frequency. We observed that the binding stabilization between spike and antibodies was more affected by the substitutions of positively charged and hydrophobic amino acids. Furthermore, the *IES* was compared with experimentally measured neutralization titers and showed a high correlation (Pearson’s *r* = −0.80 on average) with neutralization titers data^23,29,30^. Our findings highlight the importance of the immune-escaping hot spots and mutations on the RBD. These results demonstrated that our approach and identified immune-escaping hot spots can suggest a high immune-escaping epitope map for use in current vaccine design strategies and provide a rationale for the development of anti-immune-escaping vaccines.

## Results

### In silico mutagenesis for delta binding free energy prediction

**Figure 1:**
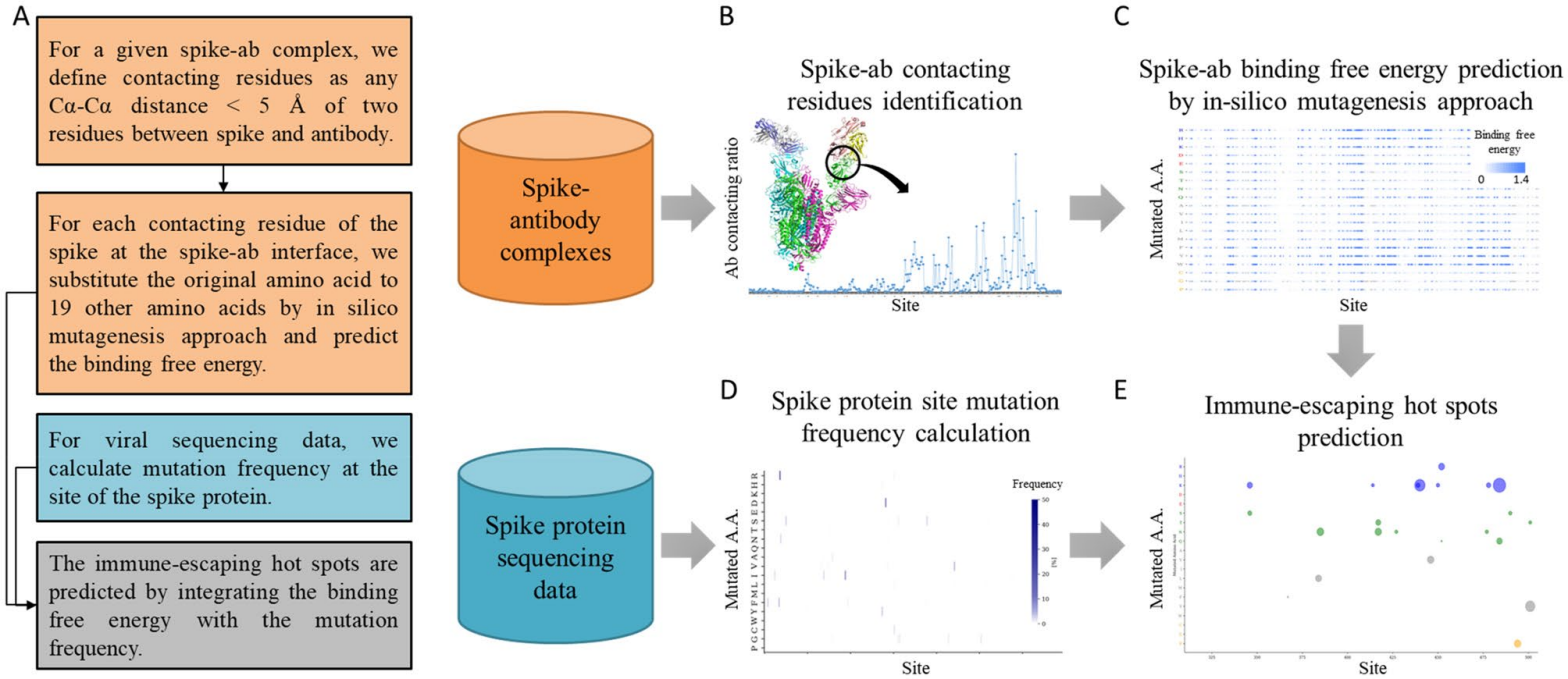
Overview of our methodology. (**A**) The overall pipeline for predicting immune-escaping hot spots. (**B**) Spike-ab contacting residues identification from protein complexes. (**C**) Spike-ab delta binding free energy estimation by in silico mutagenesis approach. (**D**) Spike protein mutation frequency calculation from viral sequencing data. (**E**) The predicted immune-escaping hot spots for suggesting and designing future vaccines.

**Figure 2:**
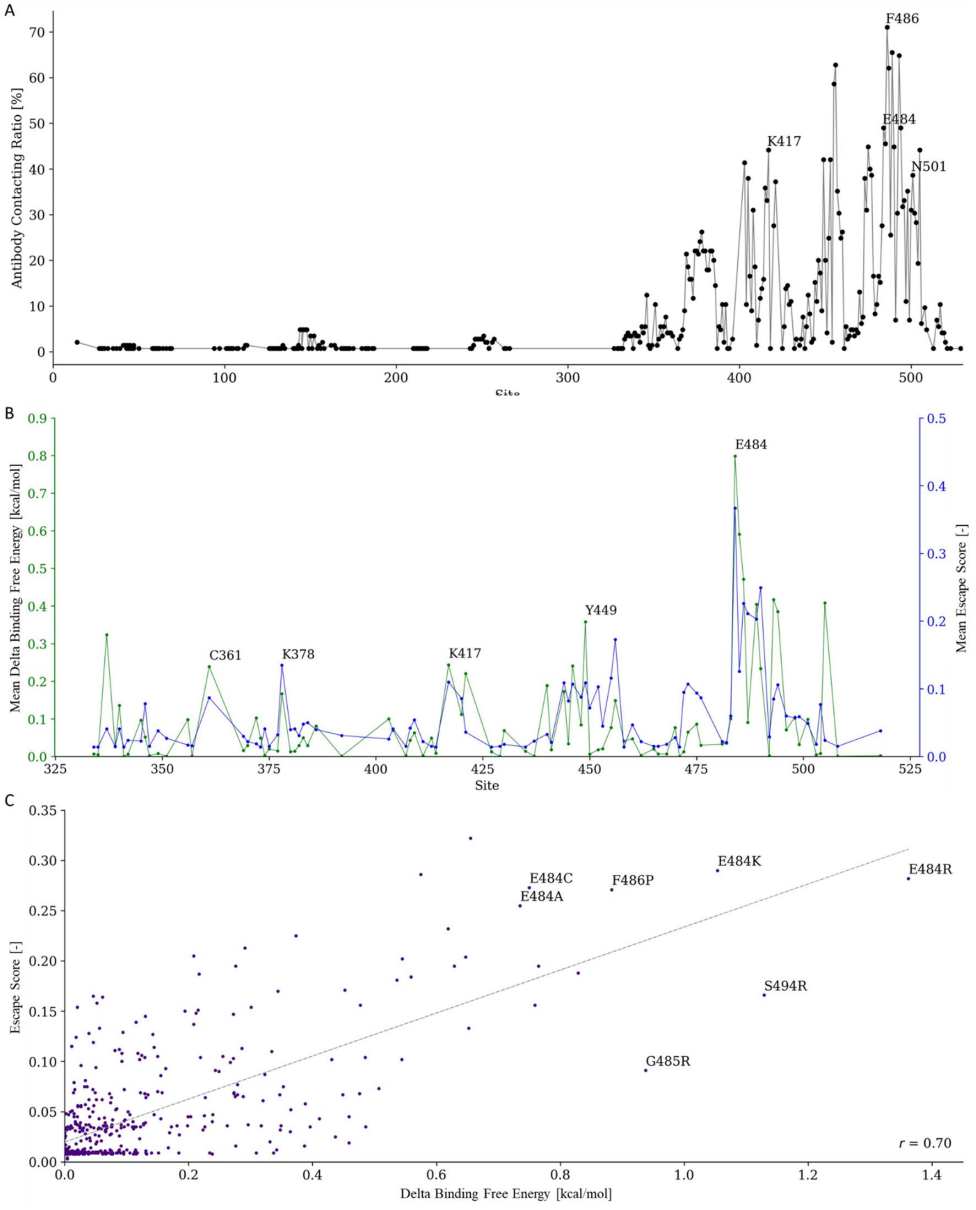
Spike-antibody contacting residue analysis and delta binding free energy estimation. (**A**) The distribution of antibodies contacting sites on spike protein. (**B**) Delta binding free energy compared with ES on spike protein sites and (**C**) the specific site mutations.

To identify spike-ab contacting residues and predict delta binding free energy by the in silico mutagenesis approach, we collected SARS-CoV-2 spike-antibody complexes from PDB^31^ (Fig. 1B, C). We noted that 94% (136/145) of antibodies were RBD-specific and only 9% in the NTD (13/145). Based on this phenomenon and limitation, we focused analysis and prediction of mutation hot spots on the RBD. The spike-antibody interacting interfaces were identified based on the Cα-Cα distance of any two residues between different chains (i.e., spike and antibody) less than 5 Å. The ratio of contacting residues between spike and antibody binding interfaces was shown in Fig. 2A. We observed that the antibodies mainly contacted (17.5% on average) the sites 346 to 517 within the RBD of the spike. The results were in line with previous studies which showed that the spike sites from 331 to 517 were the epitopes for SARS-CoV-2 neutralizing antibodies^32–34^. We observed that 103 (71%) antibodies bound to F486, implying high immunogenicity. In the most recent pandemic variants, F486 was mutated in the omicron variants. F486V, a mutation in both omicron subvariants BA.4 and BA.5, has been reported to broadly impair the neutralizing activity of several class 1 and 2 RBD monoclonal antibodies^35^. In addition, we found that 71 (49%) antibodies contacted E484, which is the mutation site that caused severe immune-escaping in omicron^36^, beta^37^, and gamma^38^ variants. These results reflected that the mutations of sites and regions on the RBD highly contacted by the antibody (e.g. F486 and E484) impair antibody recognition.

To assess the binding stability of RBD and antibodies when a mutation occurred, we estimated the delta binding free energy within 20 different amino acid substitutions via an in silico mutagenesis approach. To further validate our results, we compared our predictions with the escape score (ES) obtained from the escape estimator^39^. The ES was calculated by the experimental data from the high-throughput deep mutational scanning method^27,28^. The greater the ES of a specific site mutation, the higher the immune-escaping ability. The results showed that the mean predicted delta binding free energy of each spike position highly correlated (Pearson’s *r* = 0.70; cosine similarity = 0.80) with the mean ES (Fig. 2B). The highest mean delta binding free energy was predicted for the E484 substitutions (0.8 kcal/mol), which aligned with the highest mean ES score (0.37). In addition, the E484 mutation has been mentioned to reduce the ability of neutralization antibodies in recent reports^32–34^. We observed that five mutations with high delta binding free energy agreed with ES, including E484R (delta binding free energy = 1.36 kcal/mol; ES = 0.28), E484K (delta binding free energy = 1.05 kcal/mol; ES = 0.29), E486P (delta binding free energy = 0.88 kcal/mol; ES = 0.27), E484C (delta binding free energy = 0.75 kcal/mol; ES = 0.27), and E484A (delta binding free energy = 0.74 kcal/mol; ES = 0.26) (Fig. 2C; Additional file 1: Table S1; Additional file 2: Fig. S1). Among these mutations, E484K and E484A occurred in high immune-escaping variants, such as beta, gamma, and omicron^40^. Beta and gamma variants containing E484K were observed to be resistant to certain monoclonal antibodies, as well as the E484A mutation appears to be a key contributor to the strong evasion of the antibodies in the omicron sub-lineage variants^40^. Moreover, in contrast to the results of ES, we predicted a high immune-escaping ability for S494R (delta binding free energy = 1.13 kcal/mol; ES = 0.17) and G485R (delta binding free energy = 0.94 kcal/mol; ES = 0.09) (Fig. 2C; Additional file 1: Table S1; Additional file 2: Fig. S1). S494R was reported as the potential escape risk mutation^41^, and G485R also caused decreases in neutralization titer^33^. Our results suggested previously unnoticed immune-escaping mutations that need to be concerned.

### Mutation frequency analysis for high-risk hot spots identification

**Figure 3:**
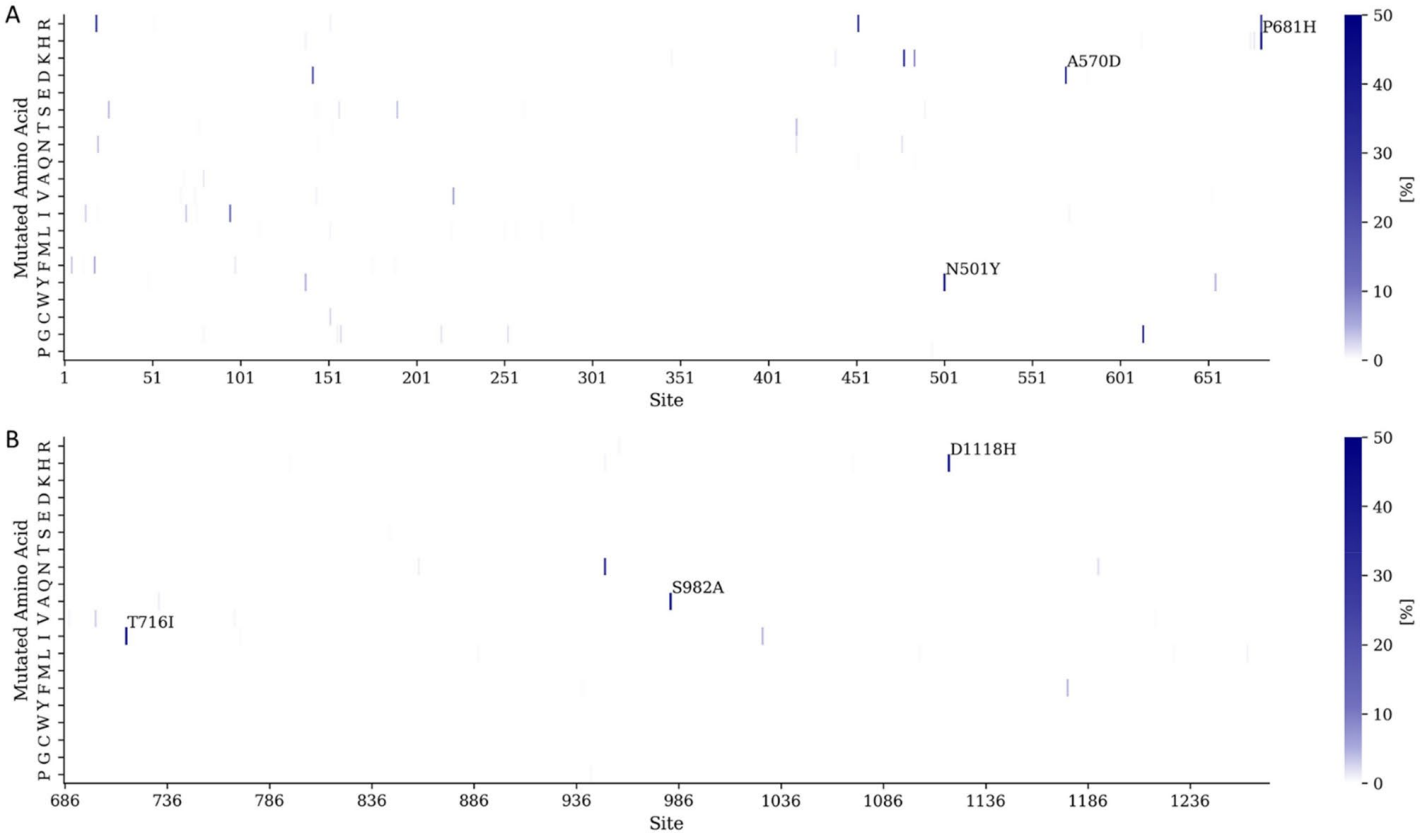
Sequenced spike protein analysis. (**A**) The heat map of mutation frequency of spike protein sites with mutated amino acids on S1 and (**B**) S2 domains.

**Figure 4:**
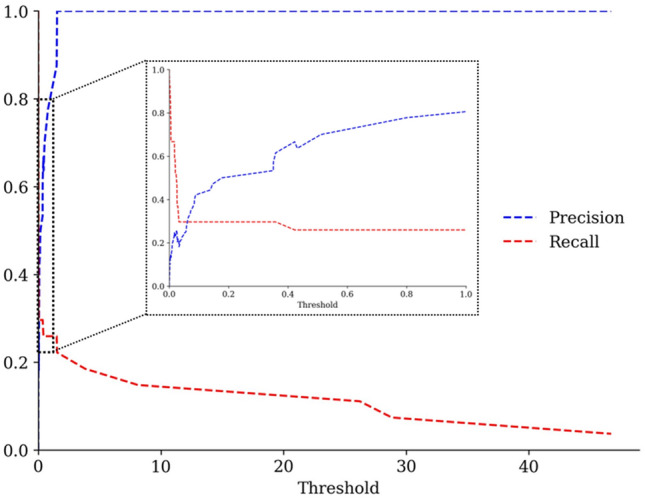
The precision and recall curve. The threshold estimations of mutation frequency.

To identify the mutation hot spots associated with pandemics, we analyzed 1,938,659 spike protein sequences of SARS-CoV-2 obtained from GISAID^42^ (Fig. 1D). Since the mutation frequency of D614 was close to 100%, we excluded it from further analysis. We observed that the mutation frequency of 6 sites on the spike protein exceeded 40%, namely the substitutions N501Y (46.7%), A570D (41.6%), P681H (44.2%), T716I (41.5%), S982A (40.5%) and D1118H (40.8%) (Fig. 3A, B). These six substitutions agreed with the substitutions found in VOCs, such as alpha and omicron variants^19^. The N501Y substitution was shown to increase the transmission of the alpha variant^20^. The A570D substitution was suggested to modulate the conformational transition of the RBD between its open and closed state^43^. The P681H substitution contributed to an increased central cavity, causing the mutated protein to be less compact^44^. The location of the D1118H substitution was suggested to potentially have an impact on the trimer assembly structure, stability, or dynamics^45^. Further, some evidence suggested that the N501Y substitution can reduce neutralization by specific RBD antibodies, highlighting its role as an escape mechanism for certain RBD antibodies^19,21,46^. Similarly, the spike protein with P681H substitution showed that escapes interferon-induced transmembrane protein (IFITM) restriction and lead to resist innate immune mechanisms^47^. Thus, the results demonstrated that our identified mutation hot spots were related to viral transmissibility, transmission, and immune-escaping ability. Furthermore, to identify the potentially high-risk hot spots that will occur in the future, we then used the mutations in VOCs (e.g., alpha and delta) and VOIs (e.g., epsilon and lambda), announced by WHO in July 2021, as the positive set. Based on these results, we considered that when the mutation frequency exceeded 0.06% that the mutation site was a high-risk hot spot (Fig. 4).

### Immune-escaping hot spots prediction

**Figure 5:**
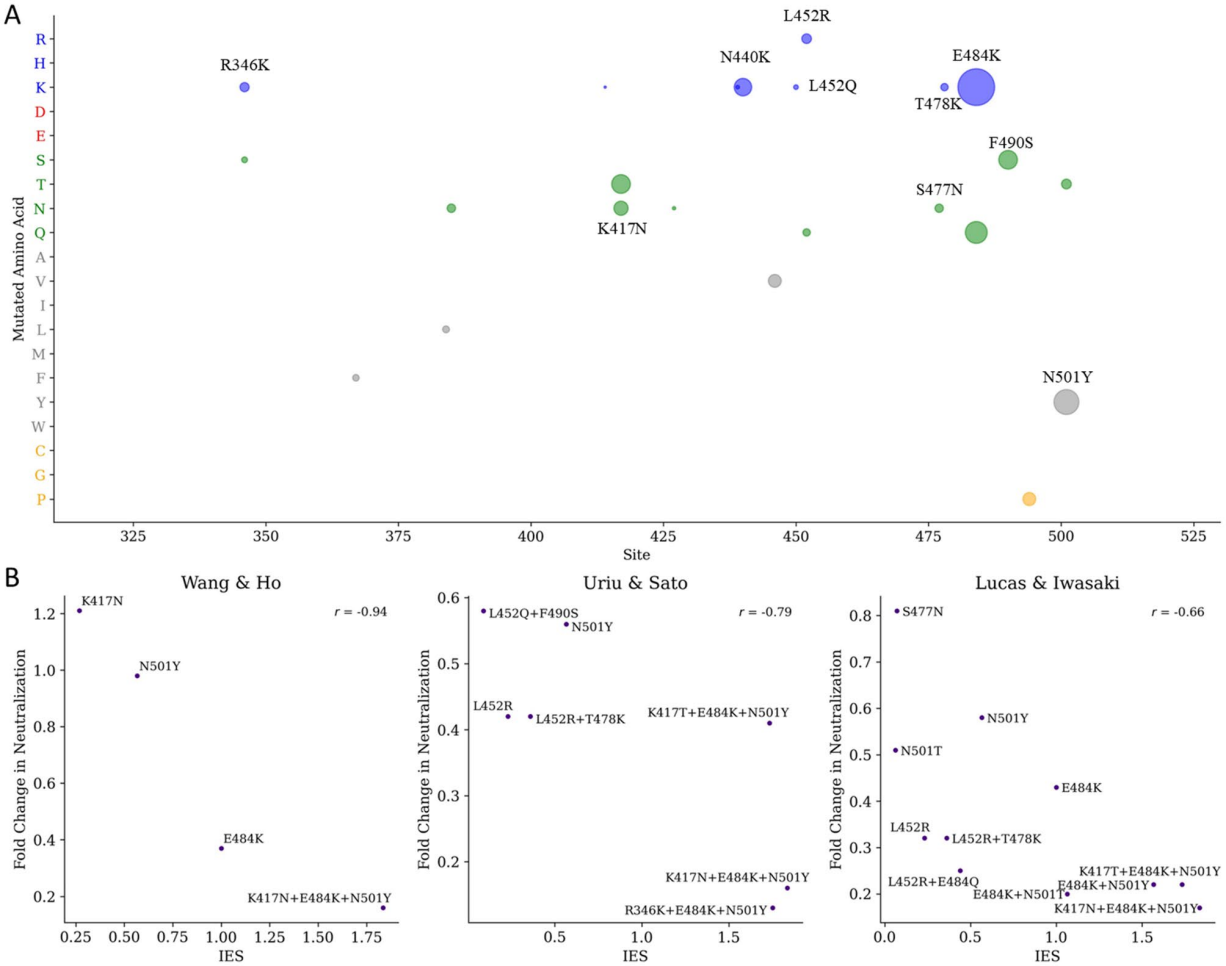
The immune-escaping hot spots on spike RBD. (**A**) The bubble plot of amino acid substitutions of contacting residues on RBD with *IES.* The colors represented the amino acid property, including blue for a positive charge, red for a negative charge, green for polar, gray for non-polar, and yellow for remains. The bubble sizes represented the strong to the weak immune-escaping ability of the *IES*. (**B**) The *IES* compared with neutralization titer data. The fold change is the geometric means of all subjects.

**Figure 6:**
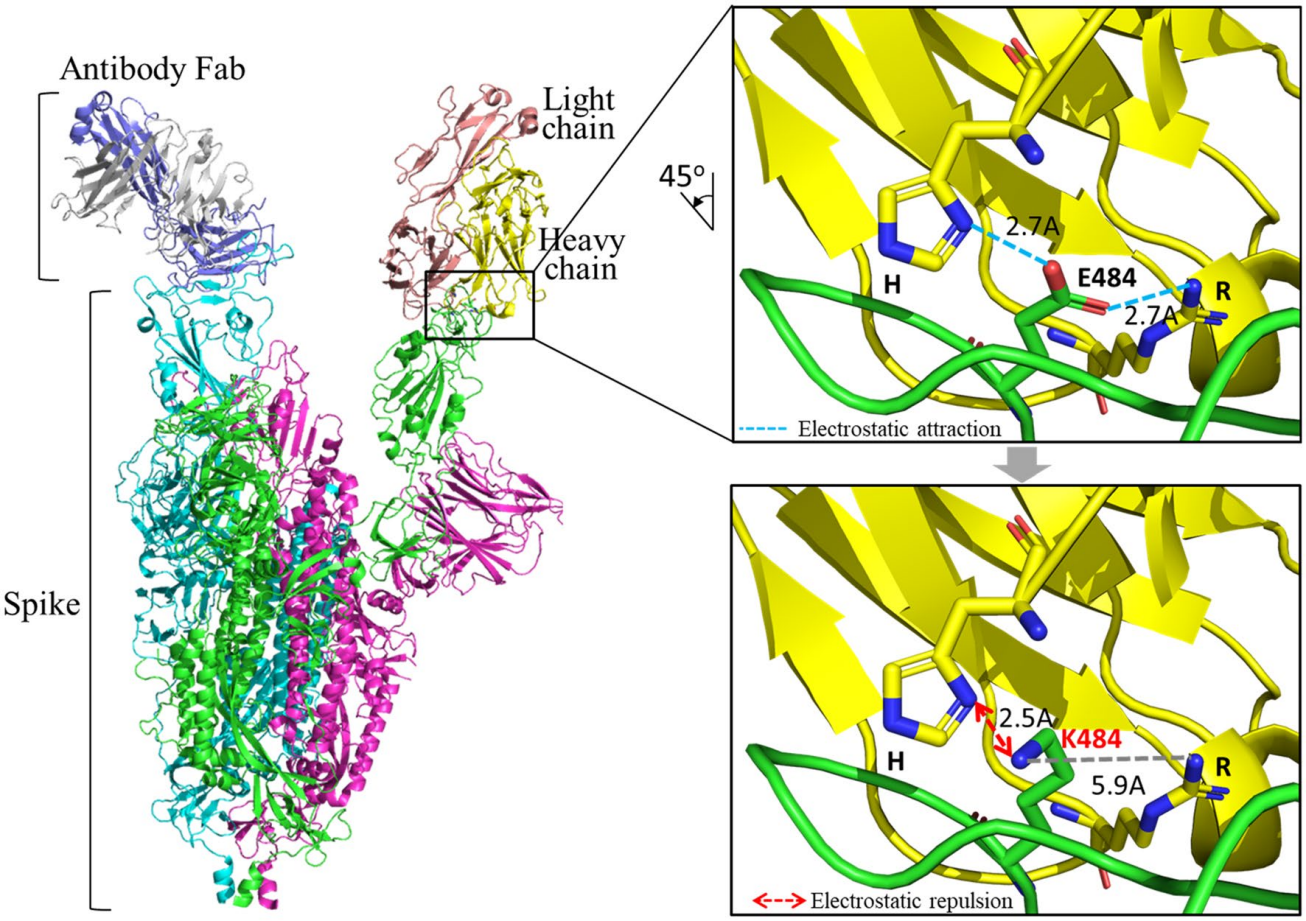
The binding mechanism of spike E484K mutation with antibody. The template of the spike-antibody complex used here was PDB ID 7DK6, and the simulation of amino acid substitution by the in silico mutagenesis method.

We integrated the delta binding free energy and the mutation frequency to calculate the *IES* to predict the immune-escaping hot spots (Fig. 1E; Eq. 6). In this paper, we identified 23 immune-escaping hot spots that had both a high delta binding free energy and a high mutation frequency (Fig. 5A; Additional file 2: Table S2). It is worth noting that nine of these predicted mutations (R346K, K417N, N440K, L452Q, L452R, S477N, T478K, F490S, and N501Y) occurred in omicron variants, despite only using data collected between January 2020 and July 2021, before the omicron first appeared in November 2021. In addition, we observed that the binding stabilization between spike and antibodies was more affected by the substitutions of positively charged and hydrophobic amino acids (Additional file 2: Fig. S1). In particular, the E484K had the highest immune-escaping ability (*IES* = 1) with a delta binding free energy of 1.05 kcal/mol and a mutation frequency of 8%. Based on the spike-antibody complex analysis, the E484K mutation converted the binding environment from a negative to a positive charge, which disrupted the interaction between the RBD and antibodies. The E484 mainly interacted with R53 and H102 in the antibody and contributed electrostatic force (Fig. 6). After the mutation to K484, the shortest distance between the oxygen atom in Lysine (K) and the nitrogen atom in Arginine (R) changed from 2.7 to 5.9 Å, leading to the interaction disappearing. The electrostatic force was converted to the unstable repulsion force from −13.7 to 97.82 cal/mol. This result agreed with recent reports that the E484K mutation significantly reduced the ability of neutralization antibodies^32–34^.

Furthermore, to assess how the *IES* compares to experimentally measured neutralization titers, we collected antibody neutralization data from three previously published studies^23,29,30^ (Fig. 5B). The results showed a high correlation between the *IES* and neutralization titers data from Wang^23^ (Pearson’s *r* = −0.94), Uriu^29^ (Pearson’s *r* = −0.79), and Lucas^30^ (Pearson’s *r* = −0.66). These results suggested that our identified hot spots can reflect the clinically observed phenomena of the SARS-CoV-2 mutations that caused immune escape. We can provide a high immune-escaping epitope map of the RBD, which can use in current vaccine design strategies, including heterologous prime-boost vaccination regimens, construction of chimeric immunogens, and design of protein nanoparticle antigens^48^. Our findings highlight the importance of the immune-escaping hot spots of the RBD in the design of future Covid-19 vaccines and provide a rationale for the development of anti-immune-escaping vaccines through the induction of antibodies against the RBD.

## Discussion

Since SARS-CoV-2 first appeared, considerable evolution has taken place such as alpha, delta, and recent omicron variants. These VOCs have shown evidence of higher transmissibility and immune-escaping ability^19–22^. The VOCs contained certain key mutations, such as E484K and N501Y, that were located on the RBD of the spike protein. Some mutations also occurred at an antigenic supersite of an N-terminal domain NTD^23,24^. Both RBD and NTD are the targets of potent virus-neutralizing antibodies against the spike protein. As SARS-CoV-2 continues to evolve and mutate, the arising of new VOCs cannot be prevented, and the alterations of the RBD and NTD in the spike protein reduce the effectiveness of currently used vaccines. Thus, developing future vaccines to protect against future mutations and variants remains crucial.

To facilitate the design of future COVID-19 vaccines, here, we proposed the computational approach by integrating the delta binding free energy measured by computational mutagenesis of spike-antibody complexes and the mutation frequency calculated from viral genome sequencing data to predict immune-escaping mutations on the RBD of the spike protein. To validate our predicted results, we used the ES data, which was obtained from the escape estimator and calculated by the experimental data from the high-throughput deep mutational scanning method^27,28,39^. The results showed that the mean delta binding free energy of each spike position highly correlated (Pearson’s *r* = 0.70) with the ES data. The highest mean delta binding free energy was predicted for the E484 substitutions (0.8 kcal/mol), which were associated with the highest mean ES score of 0.37. This suggests that the substitutions at position 484 are less tolerated on the RBD, meaning they are more likely to disrupt the protein's structure and function. In addition, the E484 mutation has been mentioned to reduce the ability of neutralization antibodies in recent reports^32–34^. We identified five E484 mutations that were consistent with ES data, including E484R (delta binding free energy = 1.36 kcal/mol; ES = 0.28), E484K (delta binding free energy = 1.05 kcal/mol; ES = 0.29), E486P (delta binding free energy = 0.88 kcal/mol; ES = 0.27), E484C (delta binding free energy = 0.75 kcal/mol; ES = 0.27), and E484A (delta binding free energy = 0.74 kcal/mol; ES = 0.26). Two of these mutations, E484K and E484A, are found in high immune-escaping variants of the virus. These variants belong to the beta, gamma, and omicron variants and are known for their ability to partially evade the immune response, potentially leading to reinfections or reduced vaccine efficacy. We also predicted high immune-escaping risk for S494R (delta binding free energy = 1.13 kcal/mol; ES = 0.17) and G485R (delta binding free energy = 0.94 kcal/mol; ES = 0.09), which were previously unnoticed immune-escaping mutations. These mutations may lead to alterations in the protein structure that could allow the virus to partially evade the host immune response, making it more challenging for the immune system to recognize and neutralize the virus effectively.

For the analysis of spike protein sequences, we observed that the mutation frequency of six sites on the spike protein exceeded 40%, such as N501Y and P681H. There is some evidence to suggest that N501Y can reduce neutralization by certain antibodies that target the RBD of the spike protein^19,21,46^. The P681H mutation has been associated with the ability to escape IFITM restriction and resist innate immune mechanisms^47^. The results demonstrated that our identified mutation hot spots were related to viral transmissibility, transmission, and immune-escaping ability. In addition, these substitutions agreed with the mutations found in the alpha and omicron variants^19^.

We then integrated the delta binding free energy and the mutation frequency to estimate the *IES* for predicting the immune-escaping hot spots. Here, we identified 23 mutation hot spots that had both a high delta binding free energy and a certain degree of mutation frequency. Although the data used for the analysis were collected from January 2020 to July 2021, before the emergence of the omicron variant in November 2021, the method successfully predicted nine mutations (R346K, K417N, N440K, L452Q, L452R, S477N, T478K, F490S, and N501Y) that were later observed in the omicron variants. Among these mutations, we observed that the binding stabilization between spike protein and antibodies was more affected by the substitutions of positively charged and hydrophobic amino acids. For instance, the E484K mutation (*IES* = 1) resulted in the change in the binding environment from a negative charge (E) to a positive charge (K) between the RBD and the antibodies. This alteration disrupted the interaction between the RBD and antibodies, potentially making it more challenging for antibodies to bind and neutralize the virus effectively.

Furthermore, the *IES* was compared with experimentally measured neutralization titers. Our results showed that the *IES* correlated highly with neutralization titers data from Wang^23^ (Pearson’s *r* = −0.94), Uriu^29^ (Pearson’s *r* = −0.79), and Lucas^30^ (Pearson’s *r* = −0.66). The moderate to strong correlations between *IES* and neutralization titers suggest that the proposed approach for identifying immune-escaping hot spots could be valuable for anti-immune-escaping vaccine design. By targeting these hot spots and understanding how mutations impact neutralization, we may be able to design more effective vaccines that can better combat viral variants and reduce the risk of immune escape.

Our approach has several limitations and challenges. First, the predicted immune-escaping hot spots still need to be experimentally validated. Second, one potential limitation is that our in silico mutagenesis approach relies on the quality and the number of virus-antibody structural templates, as the lower template quality may affect the accuracy of calculated binding free energy. Third, our immune-escaping ability prediction is limited by monoclonal antibody structural complexes. Predicting the immune-escaping capability of polyclonal antibodies will require further relevant data sources. Fourth, whether the spike is relatively stable or will change significantly over time still need to pay attention to, this is an ongoing and complex area of study. Unfortunately, the number of crystal structures of spike protein with different variants and antibodies is still limited to use in our analysis. Besides the crystal structures, the *IES* consider the mutation rates of spike protein, so using the viral sequencing data at different time point will change the score. As more data on different variants become available in the near future, a better understanding of how these factors change over time will be critical for staying ahead of viral evolution and ensuring effective responses to emerging variants.

## Methods

### Overview

The overall pipeline for identifying immune-escaping hot spots was shown in Fig. 1. We collected SARS-CoV-2 spike-antibody complexes from the Protein Data Bank (PDB)^31^ for the identification of spike-ab contacting residues and prediction of delta binding free energy by an in silico mutagenesis approach (Fig. 1B, C). To calculate the spike protein mutation frequency (Fig. 1D), we used sequenced strains of SARS-CoV-2 obtained from the Global Initiative on Sharing All Influenza Data (GISAID)^42^. To find the immune-escaping hot spots (Fig. 1E), we then integrated the computational results of the delta binding free energy with the mutation frequency.

### Dataset

SARS-CoV-2 spike-monoclonal antibody complexes were collected from PDB^31^ with a release date before July 2021. The query criteria included “spike” and “fab” in full text, “severe acute respiratory syndrome coronavirus 2” in the source organism, and refinement resolution less than 4.0 Å. Based on these criteria, we collected 145 spike-antibody complexes for analysis in this paper. 1,938,659 SARS-CoV-2 sequences were obtained from GISAID^42^ from January 2020 to July 2021. Here, we only selected the spike protein sequences for analysis.

### Contacting residues identification and amino acid substitution

For a given protein complex (e.g., spike-antibody) from PDB^31^, we extracted the 3D coordinates of the heavy atoms, including x, y, and z. We defined any Cα-Cα distance (i.e., Euclidean distance) of two residues between spike and antibody less than 5 Å as contacting residues. Based on the identified contacting residues of the spike protein, we further predicted the delta binding free energy of the substitution to the other 19 amino acids by our developed in silico mutagenesis approach. For the amino acid substitution on the protein complex, the side-chain orientation was predicted by the method SCWRL4^49^. We then inferred the coordinates of specific residues for all collected spike-ab complexes.

### Binding free energy estimation

In this paper, we calculated delta atomic binding free energies of contacting residues in spike-antibody complexes before and after amino acid substitution on spike protein by using empirical force fields derived from previous work^50^. We modified the energy function to estimate the Van der Waals, hydrogen bonds, π–π stacking, and electrostatic forces between two atom pairs. The energy function was defined as:

1 $${E}_{total}={E}_{vdw}+{E}_{hb}+{E}_{pi}+{E}_{elec}$$

where *E*_*vdw*_, *E*_*hb*_, *E*_*pi*_, and *E*_*elec*_ are the Van der Waals forces, hydrogen bonds, π-π stacking interactions, and electrostatic forces, respectively. The energy function of the pairwise atoms for the Van der Waals interactions was given as:

2 $$ E_{{vdw}}  = \left\{ {\begin{array}{*{20}l}    {P_{{vdw\_5}}  - \frac{{P_{{vdw\_5}} r_{{ij}} }}{{P_{{vdw\_1}} }},} \hfill & {if~~~~r_{{ij}}  \le P_{{vdw\_1}} } \hfill  \\    {\frac{{P_{{vdw\_6}} \left( {r_{{ij}}  - P_{{vdw\_1}} } \right)}}{{P_{{vdw\_2}}  - P_{{vdw\_1}} }},} \hfill & {if~~P_{{vdw\_1}}  < r_{{ij}}  \le P_{{vdw\_2}} } \hfill  \\    {P_{{vdw\_6}} ,} \hfill & {~if~P_{{vdw\_2}}  < r_{{ij}}  \le P_{{vdw\_3}} } \hfill  \\    {P_{{vdw\_6}}  - \frac{{P_{{vdw\_6}} \left( {r_{{ij}}  - P_{{vdw\_3}} } \right)}}{{P_{{vdw\_4}}  - P_{{vdw\_3}} }},} \hfill & {if~P_{{vdw\_3}}  < r_{{ij}}  \le P_{{vdw\_4}} } \hfill  \\    {0,} \hfill & {~if~r_{{ij}}  > P_{{vdw\_4}} } \hfill  \\   \end{array} } \right. $$

*r*_*ij*_ is the distance between the atoms *i* and *j* forming the pairwise heavy atoms between proteins. The parameters, *P*_*vdw_1*_ to *P*_*vdw_6*_, for estimating Van der Waals forces in different atom-pair distances were 3.0, 3.6, 4.5, 6.0, 20, and −0.3 Å, respectively. The energy contributed by hydrogen bonds is larger than the Van der Waals force. Here, the atom is classified into three different atom types, namely donor, acceptor, and both. A heavy atom that was a primary or secondary amine or sulfur was defined as a donor. A heavy atom that was oxygen or nitrogen with no bound hydrogen was defined as an acceptor. The heavy atom with hydroxyl group was defined as both (i.e., donor and acceptor). A hydrogen bond was able to be formed by the following atom-pair types: donor–acceptor, donor-both, acceptor-both, and both-both. The hydrogen bond energy was calculated by the following scoring functions:

3 $$ E_{{hb}}  = \left\{ {\begin{array}{*{20}l}    {P_{{hb\_5}}  - \frac{{P_{{hb\_5}} r_{{ij}} }}{{P_{{hb\_1}} }},} \hfill & {if~r_{{ij}}  \le P_{{hb\_1}} } \hfill  \\    {\frac{{P_{{hb\_6}} \left( {r_{{ij}}  - P_{{hb\_1}} } \right)}}{{P_{{hb\_2}}  - P_{{hb\_1}} }},} \hfill & {if~P_{{hb\_1}}  < r_{{ij}}  \le P_{{hb\_2}} } \hfill  \\    {P_{{hb\_6}} ,} \hfill & {~~if~P_{{hb\_2}}  < r_{{ij}}  \le P_{{hb\_3}} } \hfill  \\    {P_{{hb\_6}}  - \frac{{P_{{hb\_6}} \left( {r_{{ij}}  - P_{{hb\_3}} } \right)}}{{P_{{hb\_4}}  - P_{{hb\_3}} }},} \hfill & {~if~P_{{hb\_3}}  < r_{{ij}}  \le P_{{hb\_4}} } \hfill  \\    {0,} \hfill & {if~r_{{ij}}  > P_{{hb\_4}} } \hfill  \\   \end{array} } \right. $$

The parameters, *P*_*hb_1*_ to *P*_*hb_6*_, for estimating hydrogen bond energies in different atom-pair distances were 2.3, 2.6, 3.1, 3.6, 20, and −2.5 Å, respectively. The π–π stacking interactions were formed by aromatic residues, such as phenylalanine (F), tryptophan (W), and tyrosine (Y). The energy of π–π stacking interactions was defined as the following:

4 $$ E_{{pi}}  = \left\{ {\begin{array}{*{20}l}    {P_{{pi\_5}}  - \frac{{P_{{pi\_5}} r_{{ij}} }}{{P_{{pi\_1}} }},} \hfill & {if~r_{{ij}}  \le P_{{pi\_1}} } \hfill  \\    {\frac{{P_{{pi\_6}} \left( {r_{{ij}}  - P_{{pi\_1}} } \right)}}{{P_{{pi\_2}}  - P_{{pi\_1}} }},} \hfill & {~if~P_{{pi\_1}}  < r_{{ij}}  \le P_{{pi\_2}} } \hfill  \\    {P_{{pi\_6}} ,} \hfill & {if~P_{{pi\_2}}  < r_{{ij}}  \le P_{{pi\_3}} } \hfill  \\    {0,} \hfill & {if~r_{{ij}}  > P_{{pi\_3}} } \hfill  \\    \end{array} } \right. $$

The parameters, *P*_*pi_1*_ to *P*_*pi_6*_, for estimating hydrogen bond energies in different atom-pair distances were 3.2, 3.6, 4.5, 5.2, 20, and −0.3 Å, respectively. The electrostatic force was defined as:

5 $${E}_{elec}=332\frac{{q}_{i}{q}_{j}}{{4r}_{ij}^{2}},\quad if \,\,0.5<{r}_{ij}\le 8$$

where *q*_*i*_ and *q*_*j*_ are the formal charges, and 332 is a constant value that converts the electrostatic energy into kilocalories per mole (kcal/mol). The *r*_*ij*_ was defined as 0.5 Å, if the atom-pair distance was less than 0.5 Å, and defined as 0 Å for distances greater than 8 Å. The formal charge of the atom was defined as 0.5 for the N atom in the ND1 and NE2 of histidine and the NH1 and NH2 of arginine, −0.5 for the O atom in the OD1 and OD2 of aspartic acid and the OE1 and OE2 of glutamic acid, 1 for the N atom in the NZ of lysine, and 0 for all other atoms.

### Immune-escaping score calculation

To predict immune-escaping hot spots, we integrated the delta binding free energy and mutation frequency to calculate the immune-escaping score (*IES*), which was defined as follows:

6 $$IES=\Delta E\times F$$

where $\Delta E$ was the mean of delta binding free energy before and after amino acid substitution on spike protein within 145 spike-antibody complexes, and *F* was the mutation frequency. We used the mutations in VOCs (e.g., alpha and delta) and VOIs (e.g., epsilon and lambda), announced by WHO in July 2021, as the positive set to estimate the cut-off for identifying potential mutations that will occur in the future. Based on the precision-recall versus threshold curve, we set *F* to 1 if the mutation frequency exceeded 0.06%, else to 0 (Fig. 4). Finally, the *IES* was rescaled from 1 to 0, representing vital to weak immune-escaping ability, using the min–max normalization.

### Supplementary Information

Supplementary Table S1.

Supplementary Information.

## Data Availability

The data supporting this study’s findings are available from the corresponding author upon reasonable request.
