# Supplementary material for: In silico prediction of immune-escaping hot spots for future COVID-19 vaccine design
Source: Sci Rep. 2023 Aug 18;13:13468. doi: 10.1038/s41598-023-40741-1 (PMC10439115; doi:10.1038/s41598-023-40741-1)
Supplement: Supplementary file 2 — Supplementary Information. [file 41598_2023_40741_MOESM2_ESM.docx]

**In silico prediction of immune-escaping hot spots for future COVID-19 vaccine design**

**(Supplementary Material)**

Sing-Han Huang^1*^, Yi-Ting Chen^1^, Xiang-Yu Lin^1^, Yi-Yi Ly^1^, Ssu-Ting Lien^1^, Pei-Hsin Chen^1^, Cheng-Tang Wang^1^, Suh-Chin Wu^2^, Chwen-Cheng Chen^2^ and Ching-Yung Lin^1*^

^1^Graphen Inc., New York, NY 10110, USA.

^2^Adimmune Corp., Taichung City 427003, Taiwan

*Correspondence: johnhuang@graphen.ai (S.H.H.); cylin@graphen.ai (C.Y.L.)

**Table of Contents**

**I. Additional Figure**

Figure S1. The distribution of delta binding free energies on spike RBD.

**II. Additional Table**

Table S2. The list of predicted 23 immune-escaping hot spots.

**I. Additional Figure**


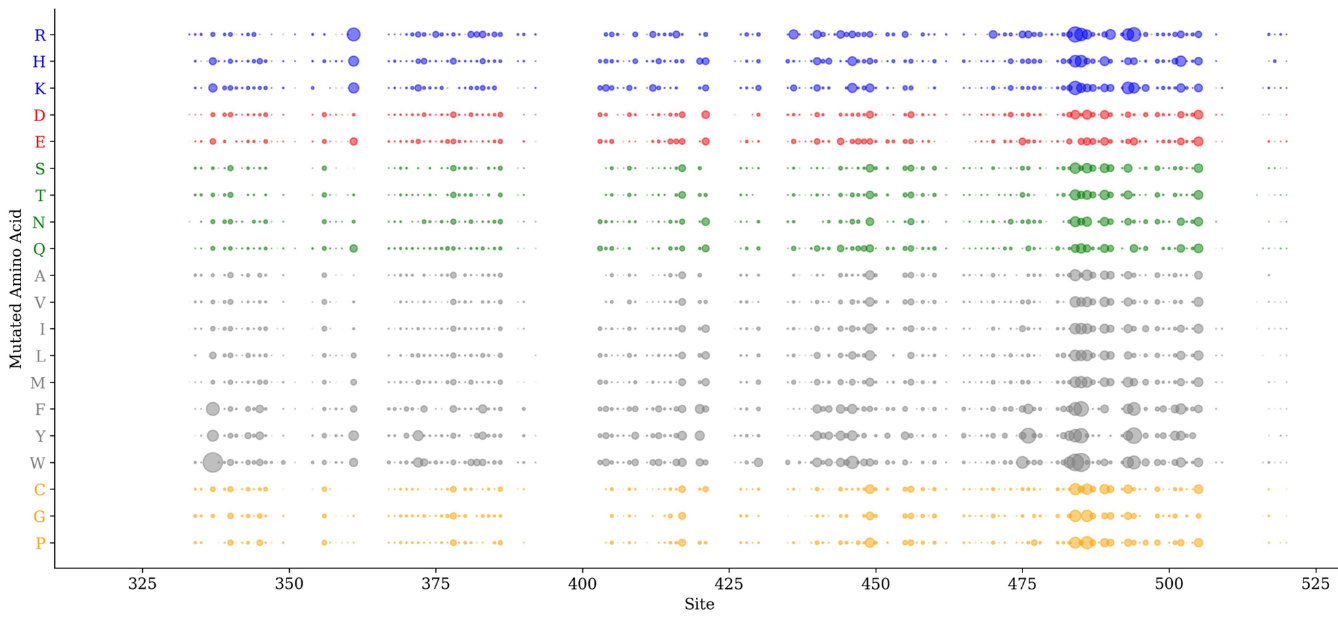


**Figure S1. The distribution of delta binding free energies on spike RBD.** The bubble plot of amino acid substitutions of contacting residues on RBD with delta binding free energy**.** The colors represented the amino acid property, including blue for a positive charge, red for a negative charge, green for polar, gray for non-polar, and yellow for remains. The bubble sizes represented the delta binding free energy from high to low.

**II. Additional Table**

| **Wild type** | **Site** | **Substitution** | **Delta binding free energy (**$\boldsymbol{\Delta E}$**)** | **Mutation frequency (*F*)** | ***IES*** | **Omicron variants** |
| --- | --- | --- | --- | --- | --- | --- |
| E | 484 | K | 1.054 | 0.084 | 1.000 |  |
| N | 501 | Y | 0.486 | 0.624 | 0.461 | Y |
| E | 484 | Q | 0.373 | 0.004 | 0.354 |  |
| K | 417 | T | 0.273 | 0.033 | 0.259 |  |
| F | 490 | S | 0.272 | 0.005 | 0.258 | Y |
| N | 440 | K | 0.239 | 0.003 | 0.227 | Y |
| K | 417 | N | 0.157 | 0.016 | 0.149 | Y |
| G | 446 | V | 0.129 | 0.001 | 0.123 |  |
| S | 494 | P | 0.129 | 0.005 | 0.122 |  |
| N | 501 | T | 0.075 | 0.001 | 0.071 |  |
| L | 452 | R | 0.072 | 0.191 | 0.069 | Y |
| R | 346 | K | 0.066 | 0.001 | 0.063 | Y |
| T | 385 | N | 0.056 | 0.001 | 0.053 |  |
| S | 477 | N | 0.054 | 0.016 | 0.051 | Y |
| L | 452 | Q | 0.043 | 0.003 | 0.040 | Y |
| T | 478 | K | 0.042 | 0.163 | 0.040 | Y |
| P | 384 | L | 0.037 | 0.001 | 0.035 |  |
| V | 367 | F | 0.033 | 0.001 | 0.031 |  |
| R | 346 | S | 0.026 | 0.001 | 0.025 |  |
| N | 450 | K | 0.017 | 0.001 | 0.016 |  |
| N | 439 | K | 0.010 | 0.007 | 0.010 |  |
| D | 427 | N | 0.009 | 0.001 | 0.008 |  |
| Q | 414 | K | 0.004 | 0.001 | 0.004 |  |

**Table S2. The list of predicted 23 immune-escaping hot spots.**
